# A Novel Amphoteric Ion-Modified, Styrene-Based Nano-Microsphere and Its Application in Drilling Fluid

**DOI:** 10.3390/ma16186096

**Published:** 2023-09-06

**Authors:** Xianfa Zhang, Jingping Liu, Jinsheng Sun, Zonglun Wang, Zhiwen Dai, Yuanwei Sun, Taifeng Zhang

**Affiliations:** 1 School of Petroleum Engineering, China University of Petroleum (East China), Qingdao 266580, China; mrzhangxianfa@163.com (X.Z.);; 2 CNPC Engineering Technology R & D Company Limited, Beijing 102206, China

**Keywords:** drilling fluid, nano-microsphere, plugging, high-temperature and high-salinity, deep oil and gas

## Abstract

With the gradual depletion of shallow oil and gas, deep oil and gas has become the focus of development. However, deep formations generally face the challenge of high-temperature and high-salinity, and drilling fluid agents are prone to failure, leading to drilling fluid intrusion into the formation that can cause serious drilling accidents such as well bore collapse. For this, a styrene-based nano-microsphere (SSD) modified with amphoteric ions was developed, with a particle size of 228 nm which could resist temperatures up to 200 °C and sodium chloride (NaCl) up to saturation. SSD has significant salt-responsive properties and its aqueous dispersion becomes transparent with increasing salinity. The SSD provided superior plugging performance in solutions containing NaCl, with a core plugging efficiency of 95.2%, and it was significantly better than the anion-modified microspheres. In addition, in drilling fluids under high temperature and high-salinity conditions, the SSD promotes particle gradation of drilling fluids and improves the zeta potential through its own plugging and synergistic effect with clay, which significantly improves the comprehensive performance of drilling fluids, such as stability, rheological performance, and filtration reduction performance. The development of SSD provides a new idea for research of high-temperature and high-salinity-resistant drilling fluid agents.

## 1. Introduction

At present, oil and natural gas are still one of the most important energy sources in the world [1,2,3]. With the continuous development of petroleum resources, shallow resources are gradually depleted, and deep ultra-deep oil and gas has become a key development object [4,5,6,7]. However, deep formations generally face complex challenges such as high-temperature and high-salinity, which requires higher drilling technology [8,9,10,11].

Drilling fluid is indispensable the drilling process, called the blood of drilling [12,13]. It flows in from the drill pipe and out in the gap between the drill pipe and the formation, and it has various effects such as carrying rock chips, protecting the well bore, balancing the formation pressure, assisting in breaking rock, etc. [14,15]. Its performance determines the success or failure of drilling. In deep drilling, the molecular chain of agents in drilling fluids will curl and break under the effect of high-temperature and high-salinity [16,17], resulting in a significant deterioration of the performance of the drilling fluid [18]. The drilling fluid will penetrate into the formation through the micropores of the sandstone and mudstone, which will easily cause the wall to collapse, and even lead to borehole failure [19]. Well bore stability and drilling safety in deep and complex formations are difficult to ensure, seriously hindering the development of deep oil and gas.

In order to prevent the drilling fluid from infiltrating and intruding into the formation under high-temperature and high-salinity conditions, and to maintain the stability of the well bore, many countries have adopted the method of plugging the pore space of the formation [19,20,21,22,23]. At present, the main categories of plugging materials are inorganic plugging materials and polymer-based plugging materials [24,25]. Inorganic materials have excellent temperature resistance and are not sensitive to salt, mainly calcium carbonate nanoparticles, silicon dioxide nanoparticles and graphene oxide [26,27,28]. However, nanomaterials are easy to aggregate and not easy to deform, resulting in poor compatibility with pores and a poor plugging effect. Polymer-based plugging materials have been one of the main research focuses in recent years because of their self-adaptability, good plugging effect, and good compatibility with drilling fluids [29,30,31]. Polymer-based plugging agents are dominated by styrene-based microspheres. Cheng et al. [32] prepared a nanoparticle by copolymerisation of styrene, butyl methacrylate, and butyl acrylate, which could effectively reduce the fluid loss in drilling fluids at 150 °C and achieve a core sealing rate of 88.75%. Li et al. [26] prepared a polymer nanosphere with a double cross-linked structure to plug shale pores, which could resist temperatures up to 200 °C in the drilling fluid. Zhong et al. [33] introduced cyclodextrin polymer microspheres into drilling fluid and found that it could control the fluid loss of drilling fluid at 200 °C. Huang et al. [34] functionalised styrene microspheres using 2-acrylamido-2-methyl-1-propanesulfonic acid and diallyldimethylammonium chloride, which resulted in a reduction of the particle size of the microspheres from 371 nm to 235 nm at 220 °C, with a change of less than 40%. The temperature resistance of polymer-based plugging agents could reach more than 200 °C. However, research on the performance of organic plugging agents under high salt conditions is still lacking, and the comprehensive performance of drilling fluids under high-temperature and high-salinity still needs to be improved.

Amphoteric polymers with a reverse polyelectrolysis effect are used in drilling fluids because of their excellent salt tolerance properties, which can significantly improve the performance of drilling fluids under high-salinity conditions [35,36,37]. Therefore, in this study, two monomers with opposite ions were introduced into styrene microspheres to prepare a plugging agent with excellent high-temperature and salt-resistant properties, and the plugging agent could significantly improve the comprehensive performance of drilling fluids. The development of this microsphere provides a new method for the research of high-temperature and salt-resistant treatment agents.

## 2. Materials and Methods

### 2.1. Materials

Sodium styrene sulfonate (SSS, 90%) and methacryloxyethyl trimethyl ammonium chloride (DMC, 75%) were purchased from McLean Biochemicals & Technologies Ltd., Shanghai, China. Styrene (ST, AR), and ammonium persulfate (AR) and sodium chloride (NaCl, AR) were purchased from Sinopharm Chemical Reagent Co., Shanghai, China. Alkylphenol ether sulfosuccinate sodium salt (MS-1, 40%) was provided by Shandong Yusuo Chemical Technology Co., Linyi, China. Bentonite is purchased from Huai’an County Tengfei Bentonite Development Co., Zhangjiakou, China.

### 2.2. Preparation of Nano-Microspheres

First, 0.2 g of MS-1 was dissolved into 80 g of deionised water and 10 g of ST, 1 g of SSS, and 1.21 g of DMC were dispersed therein sequentially, and then the dispersion was shear emulsified at 2000 r/min for 10 min. The dispersion was transferred to a flask, which was heated in a water bath at 80 °C, with a stirring speed of 700 r/min, and nitrogen was passed through to evacuate the oxygen. Ammonium persulfate, 0.05 g, was dissolved into 2 mL of deionised water and added drop by drop to the flask after stirring the dispersion for 30 min. After the reaction at constant temperature for 5 h, the resulting white emulsion is the microsphere dispersion SSD. As a comparison, microsphere emulsion SS without DMC monomer was prepared according to the same method.

### 2.3. Properties of Microspheres

The microsphere emulsions were purified by soaking them in a dialysis bag (Mw8000) in a sufficient amount of deionized water for 72 h, and then freeze-dried. The structures of the two microspheres were characterised by IR spectrometer (Shimadzu IRTracer-100, Kyoto, Japan) with scanning wavelengths ranging from 400 cm^−1^ to 4000 cm^−1^.

The microscopic morphology of the microspheres was observed with the aid of a transmission electron microscope (Tecnai G2 F20, Amsterdam, The Netherlands) after 100-fold dilution and drying by dropping on a copper grid. The particle size of the microspheres was measured by a particle size analyzer (Malvern Zetasizer Nano Z, Malvern, UK) at the same dilution.

The same mass of SSD was added to brines with different concentrations of NaCl (9 g of SSD was added to 100 mL of solution), and the transmittance of SSD dispersions at different concentrations of NaCl was investigated using a visible spectrophotometer (UV-6800A, Shanghai, China).

The plugging performance of the microspheres was evaluated using a core expulsion device (LDY50-180A, Suzhou, China). The core was made of artificial sandstone, with a length of 5 cm and a diameter of 2.5 cm. The dispersion concentration of the microsphere emulsion was 5%. The pressure transfer experiment was carried out as follows: firstly, the upstream pressure was constant at 3.5 MPa, the peripheral pressure was 5 MPa, and the back pressure was set at 3.5 MPa; then, the experiment was started and the downstream pressure was recorded. After the downstream pressure was constant, the back pressure was deflated, the maximum flow velocity was set to 2 mL/min, and the exclusion was continued for 16 h to evaluate the plugging performance.

### 2.4. Performance of SSD in Drilling Fluids

The basic drilling fluid (DF) was obtained by dispersing 16 g bentonite into 400 g of water, stirring at high speed for 20 min, and then left to stand for 24 h. SSD and NaCl etc. was dispersed into DF according to the quality requirements and stirred at high speed for 20 min to produce the required drilling fluid. Hot rolling was used to seal the drilling fluid in the aging tank tightly. With the fluid in the hot rolling furnace, it was rolled at constant temperature for 16 h; then, taken out after cooling naturally and stirring at high speed for 20 min.

The rheological properties of the drilling fluid were tested using a six-speed rotational viscometer (ZNN-D6, Qingdao, China) and a Hacker rheometer (HAAKE Mars 60, Karlsruhe, Germany), respectively.

Apparent viscosity (AV) and plastic viscosity were calculated using the formulas:

(1) $$AV=\frac{\theta_{600}}{2}(mPa\cdot s)$$

and

(2) $$PV=\theta_{600}-\theta_{300}$$

The filtration loss of drilling fluid at a differential pressure of 100 psi over 30 min was determined using a medium-pressure filtration loss meter according to API standards. The mudcake obtained from the filtration loss was dried at 105 °C and observed at 5000× (EVO LS 15, Oberkochen, Germany). The stability of the drilling fluid was tested using an emulsion stability analyzer (Turbiscan LAB Expert, Toulouse, France) with a scanning frequency of 5 min/cycle. The particle size distribution of the drilling fluid was measured using a particle size meter (Mastersizer 3000, Malvern, Britain). Zeta potential was measured by a potentiostat (Malvern Zetasizer Nano Z, UK) and the drilling fluid was diluted at 1:1000.

## 3. Results and Discussion

### 3.1. FTIR

**Figure 1:**
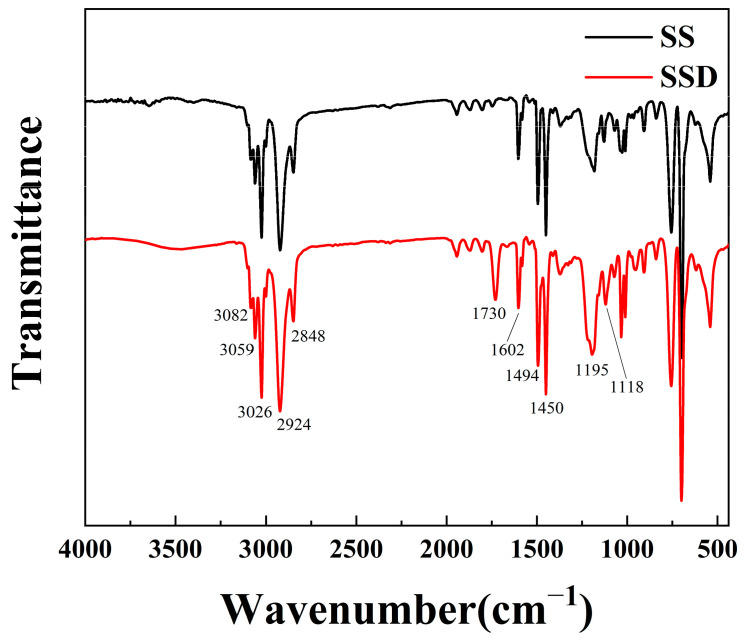
FTIR spectrum of synthesized polymeric nanospheres.

To verify the target products, the copolymers SS and SSD were characterised by FTIR as shown in Figure 1.

The FTIR spectra of the two polymeric microspheres are shown in Figure 1. The vibrational peaks at 3082 cm^−1^, 3059 cm^−1^, and 3026 cm^−1^ are C-H vibrational peaks at different positions on the benzene ring. The three C-C stretching vibrational absorption peaks in the benzene ring are at 1602 cm^−1^, 1494 cm^−1,^ and 1450 cm^−1^. The asymmetric and symmetric stretching vibrational absorption peaks of methylene in the polymer chain are found at 2926 cm^−1^ and 2833 cm^−1^. The peaks at 1195 cm^−1^ and 1118 cm^−1^ are asymmetric and symmetric stretching vibrational peaks of the sulphonic acid group, indicating that sodium styrenesulfonate was involved in the polymerization. Notably, the peak at 1730 cm^−1^ is a stretching vibration peak of C=O in the ester group, which indicates that the preparation of SSD contains DMC. In addition, there are no obvious peaks of unreacted vinyl in the spectrum, and both SS and SSD are target products and fully reacted.

### 3.2. Thermal Gravimetric Analysis

**Figure 2:**
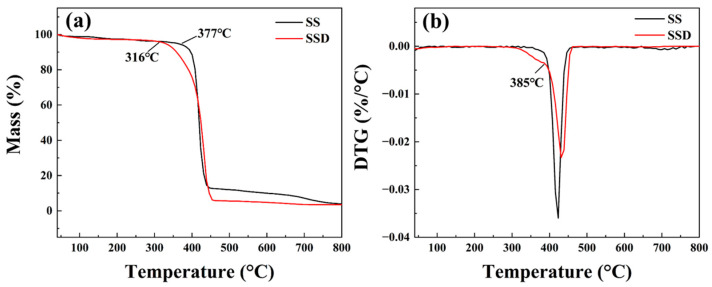
TGA (**a**) and DTG curves (**b**) of SS and SSD.

The structures of the two copolymers were further characterised by thermal gravimetric analysis, as shown in Figure 2. Under the same test conditions, the thermal decomposition temperature of SSD is lower than that of SS, but its pyrolysis temperature is still as high, at 316 °C. The cationic side chains of SSD began to undergo thermal-oxidative degradation at 316 °C, followed by a gradual decomposition of polystyrene-based spheres, and the decomposition rate increased sharply after 385 °C. The SS also has two main decomposition processes, one is the rapid decomposition of the microspheres starting at 377 °C and the other is the slow decomposition of the residual main chains and benzenesulphonic acid groups, etc., after the thermal degradation of the SS is basically completed at 430 °C. In summary, both copolymer microspheres have excellent thermal stability.

### 3.3. Morphology

**Figure 3:**
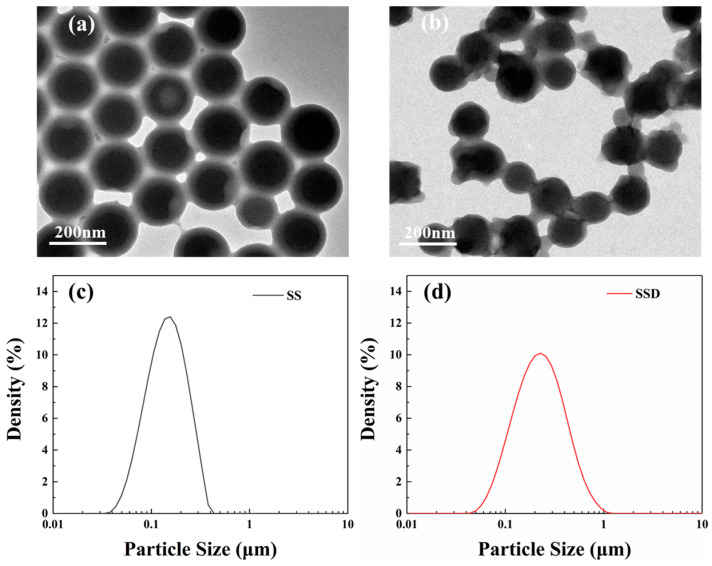
TEM images and particle size of SS (**a**,**c**) and SSD (**b**,**d**).

The morphology of the microspheres SS and SSD are shown in Figure 3a,b. SS is an anion-modified, styrene-based microsphere with a regular spherical shape and well-defined intersphere boundaries. The particle size of SS is shown in Figure 3c, with a concentrated particle size distribution and a median particle size of about 156 nm. However, with the addition of an equal amount of cation-modified monomer, i.e., SSD, the homogeneity of the microsphere size is reduced and the boundaries between the microspheres become blurred, presenting a state of interlinked small and large microspheres, as Figure 3b. As a result, in Figure 3d, the SSD shows a wider particle size distribution, with an increased median particle size of 228 nm. Interestingly, the wider particle size distribution and the presence of interspherical interactions are more conducive for the microspheres to plug the formation pores.

### 3.4. Properties of SSD in Water

#### 3.4.1. Transmittance Analysis

**Figure 4:**
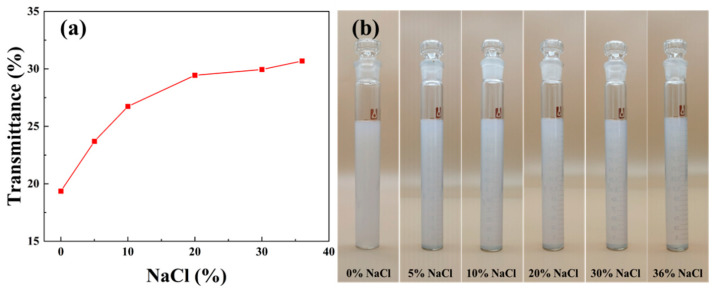
Transmittance of SSD in water with different concentrations of NaCl ((**a**) Transmittance; (**b**) appearance).

When a beam of light illuminates a medium containing dispersed particles, light transmission, light scattering, and light reflection will occur, depending on the size of the particles. If the particles of the dispersed medium are much larger than the wavelength of the incident light, light reflection occurs and the medium is opaque. Until the diameter of particles decreases to less than the wavelength of light, then light will scatter and the medium becomes transparent. When the particle size is much smaller than the wavelength, light transmission occurs and the solution is completely transparent. For SSD, its aqueous dispersion system showed different transmittance with different salt concentrations. Under the irradiation of light at 600 nm, the transmittance of SSD dispersion was improved with increasing NaCl concentration, which could increase from 19.36% to 30.68% as Figure 4a. Its state under different salinity conditions is shown in Figure 4b. The SSD dispersion is almost opaque without salt and its transparency increases with increasing salinity. At saturated NaCl (36% NaCl), it is almost transparent, resulting in the scale on the back of the cuvette being clearly visible through the SSD dispersion in room light conditions. This is due to the fact that NaCl weakens the electrostatic attraction between the polymer chains of microspheres, which increases the dispersibility and solubility of SSD and reduces the diameter of microspheres, thus, making the SSD dispersion transparent.

#### 3.4.2. Evaluation of Plugging Performance

**Figure 5:**
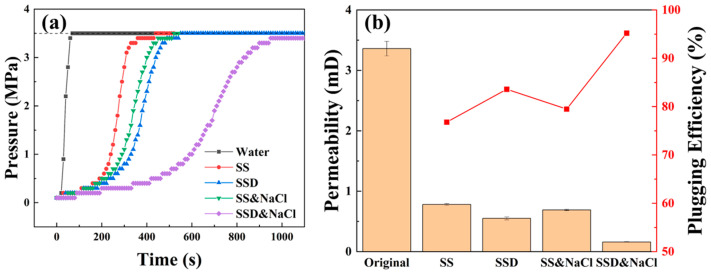
Experimental results of microspheres plugging of cores ((**a**) Pressure transmission; (**b**) Core plugging).

The plugging performance of the microspheres under different dispersion conditions was tested using core pressure transmission and core plugging experiments respectively, as shown in Figure 5. At a differential pressure of 3.5 MPa, the water flows rapidly through the core and balances the pressure at both ends of the core in a remarkably short time. When the microsphere emulsions were added to the water at a concentration of 5%, the pressure equilibration time was significantly extended. SS water dispersion, SS saturated NaCl water dispersion, and SSD water dispersion all had the effect of plugging rock pores and retarding pressure transfer in the core, but all reached pressure equilibrium within 420–550 s. However, with the addition of NaCl, the pressure transfer curve of the SSD aqueous dispersion showed a significant change and its equilibrium time improved more than twice compared to that without NaCl, and the downstream pressure still did not reach 3.5 MPa after 1100 s. The results of the core plugging experiment for 16 h are consistent with the pressure transfer experiment, as shown in Figure 5b. The SSD water dispersion containing NaCl has the optimal plugging effect, with a plugging efficiency of 95.2%. This is mainly due to the fact that, in addition to the SSD microspheres, which can plug the pores of rock by themselves, the electrostatic shielding effect of NaCl disrupts the association of polymer molecules and promotes the dissociation of positive and negative ions from the molecular chains on the surface of the microspheres, allowing the microspheres to adsorb on the rock surface to enhance the adsorption stability. Moreover, NaCl enhances the dispersion of SSD and promotes the gradation of the microspheres, which improves the plugging efficiency.

### 3.5. SSD in Drilling Fluids

#### 3.5.1. Rheological Properties

**Figure 6:**
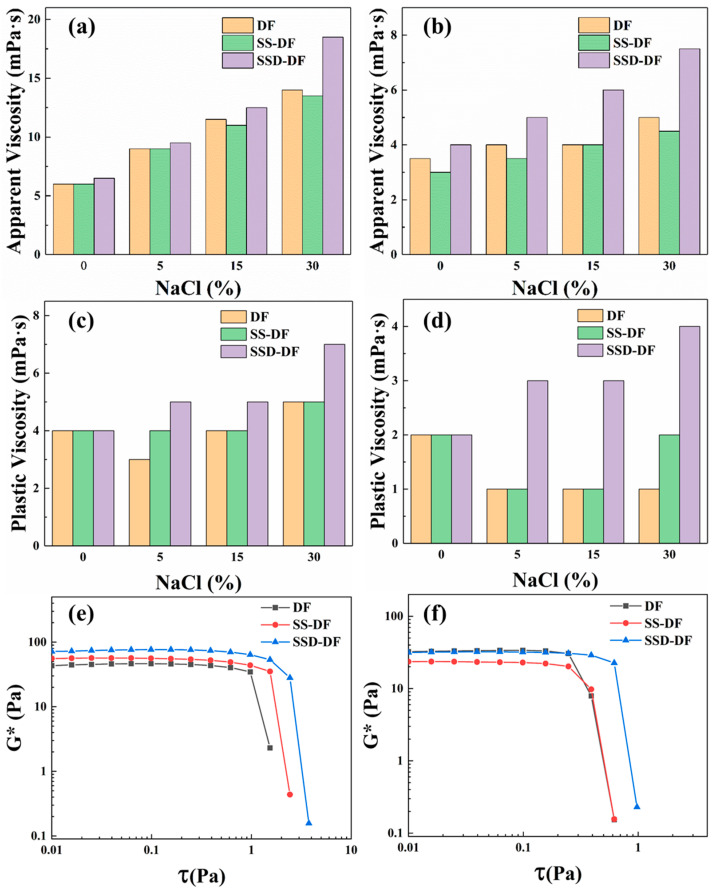
Rheological properties of drilling fluids ((**a**,**c**) 25 °C; (**b**,**d**) 200 °C; (**e**) 25 °C & 36% NaCl; (**f**) 200 °C & 36% NaCl).

The rheological properties of the drilling fluid are of critical importance. They are related to the rock-carrying properties of the drilling fluid, as shown in Figure 6. At room temperature, the apparent viscosity of DF gradually enhanced with the increase of NaCl content, which was due to the fact that NaCl compressed the diffuse double layers of clay, leading to the agglomeration of clay particles and the increase in apparent viscosity of the DF. The addition of SS failed to change the rheological properties of the drilling fluid, whereas SSD had a more pronounced viscosity-increasing effect with increasing concentration of NaCl. The high-temperature and NaCl weakened the intergranular interactions of the clay, resulting in low apparent and plastic viscosities of the clay. Even after hot rolling at 200 °C, SSD still showed a significant viscosity-increasing effect with increasing NaCl, while the added SS had no effect. This is due to the facts that NaCl brings a large number of charges to the drilling fluid, weakening the electrostatic force between positive and negative charges on the molecular chain on the surface of SSD, and the molecular chain is altered from curling to stretching, thus, improving the viscosity of the drilling fluid. In contrast, SS is an anionic monomer-modified styrene microsphere, which does not exhibit this phenomenon and ability.

Further, the effect of microspheres on the rheological properties of drilling fluids was evaluated using composite modulus (G*) tests. The composite modulus can reflect the structural strength of the reticulation in the drilling fluid, allowing the evaluation of its suspension stability and rock-carrying capacity (Sun et al., 2022). The G* in the curve are constant and then suddenly decrease, and the value of τ at this turning point represents the yield stress of the drilling fluid. Under the conditions of room temperature and saturated NaCl, both SS and SSD can increase the yield value of drilling fluids, and the ability of SSD is significantly better than that of SS. After hot rolling at 200 °C, the strength of the grid structure in the drilling fluid decreases, as does the yield value. At this time, only SSD substantially increased the yield value of DF. This indicates that SSD has excellent resistance to high-temperature and salt, which can improve the structural strength and stability of drilling fluids under the conditions of high-temperature and high-salinity.

#### 3.5.2. Fluid Loss

**Figure 7:**
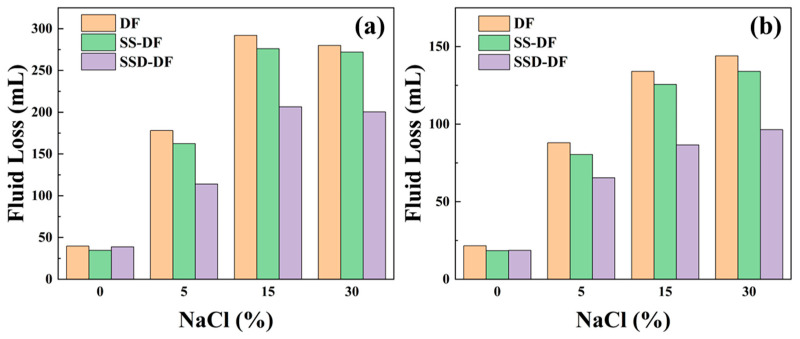
Fluid loss of drilling fluids ((**a**) 25 °C; (**b**) 200 °C).

Filtration loss reduction is another important property of drilling fluids, which is aimed at preventing water from entering the formation, maintaining the stability of the well bore and protecting the drilling safety. The drilling fluid can maintain a low fluid loss without salt, while with the addition of NaCl, the fluid loss of the drilling fluid increases abruptly. This loss of drilling fluids is caused by the destructive effect of NaCl on the diffuse double layer of clay, which leads to the reduction in the thickness of the clay hydration film, weaker interaction between particles, and reduced densification of the mud cake. High-temperature will exacerbate damage to the clay, as shown in Figure 7b. Both microspheres can reduce the fluid loss of DF, but SSD has a superior ability compared to SS. This demonstrates that the amphoteric ion-modified microspheres have better salt resistance.

#### 3.5.3. Stability of Drilling Fluids

**Figure 8:**
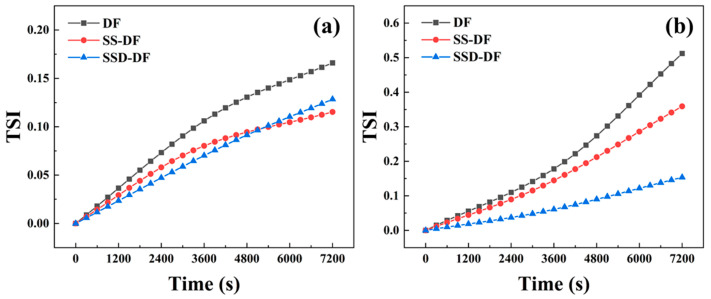
TSI of drilling fluids with saturated NaCl ((**a**) 25 °C; (**b**) 200 °C).

TSI is an evaluation of the stability of the entire dispersion system; the larger its value, the more unstable the system. Both SS and SSD can improve the stability of drilling fluids with NaCl at room temperature, as shown in Figure 8a. While the stability of drilling fluids is further reduced after high-temperature action, SSD can better maintain the dispersion and suspension stability of drilling fluids, as shown in Figure 8b, whereas SS has limited ability to do so. The clay surface shows negative electrical properties, and SS relies on the electrostatic repulsive effect of negative charges to improve the dispersion stability of the system. SSD has more significant advantages, the amphoteric ionic polymer chain on its surface is fully stretched under the action of NaCl and high-temperature and adsorbed on the surface of clay through electrostatic action, thus, participating in the construction of the grid structure in the drilling fluid and improving the stability of the drilling fluid.

### 3.6. Mechanism Analysis

#### 3.6.1. Microscopic Morphology of Mud Cake

**Figure 9:**
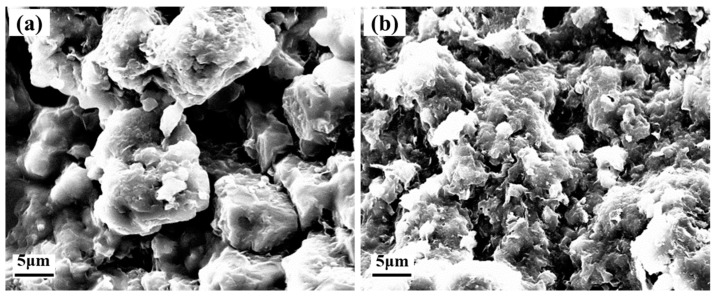
Microscopic morphology of drilling fluid mud cake under the hot rolling of 200 °C and saturated NaCl ((**a**) DF; (**b**) SSD-DF).

The microscopic morphology of the mud cake of DF after hot rolling at 200 °C and saturated NaCl is shown in Figure 9a. After the DF was hot rolled under high-temperature and high-salinity conditions, the clay particles were agglomerated and distributed in the form of large particles, and there were obvious large pores in the mud cake, which led to the drilling fluid flowing into the formation under the action of differential pressure. This is the main reason for its uncontrolled filtration. In contrast, after adding SSD to the DF, a large number of white microspheres were visible on the surface of the mud cake; SSD and clay synergistically constructed the mud cake through electrostatic action, which made a significant change in the structure of the mud cake. As shown in Figure 9b, the degree of dispersion of clay particles is enhanced, the particle size is reduced, the number and diameter of pores on the surface of the mud cake are significantly reduced, the densification of the mud cake is improved, and the fluid loss is reduced. This suggests that SSD is involved in regulating the dispersion of clay particles, in addition to plugging the mud cake pores, thus, improving the performance of the drilling fluid.

#### 3.6.2. Particle Size and Zeta Potential

**Figure 10:**
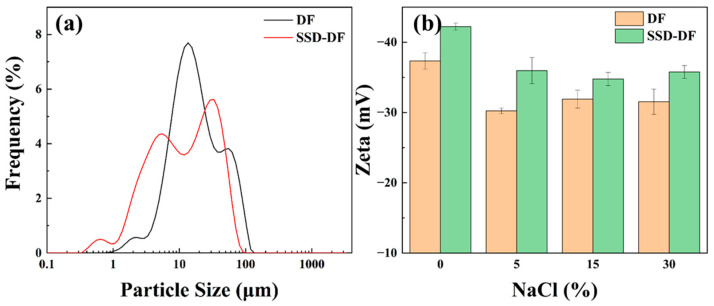
Particle size and Zeta potential of drilling fluids ((**a**) Size; (**b**) Zeta).

The mechanisms of SSD in drilling fluid was further investigated using particle size analysis and zeta potential analysis. As shown in Figure 10a, the DF particle size distribution curve shifted to the left after adding SSD, indicating that the dispersion of drilling fluid particles increased and the particle size decreased. This facilitates the formation of a denser mud cake and reduces the fluid loss of the drilling fluid. Zeta potential can reflect the stability of the system; larger absolute values of Zeta indicates a more stable the system. Poor stability of drilling fluids will lead to degradation of drilling fluids performance, solids settling, and bits burial, which is critical to drilling safety. As shown in Figure 10b, the absolute value of Zeta decreases dramatically after adding NaCl to the DF. With the increasing concentration of NaCl, the zeta values of the drilling fluids were all close to the critical value for system stability (−30 mV), and the stability of the DF was greatly reduced. Furthermore, SSD can effectively increase the absolute zeta value of DF at all NaCl concentrations, indicating that its addition enhances the stability of drilling fluid and improves the comprehensive performance of drilling fluid.

#### 3.6.3. Mechanism of SSD in Drilling Fluid

**Figure 11:**
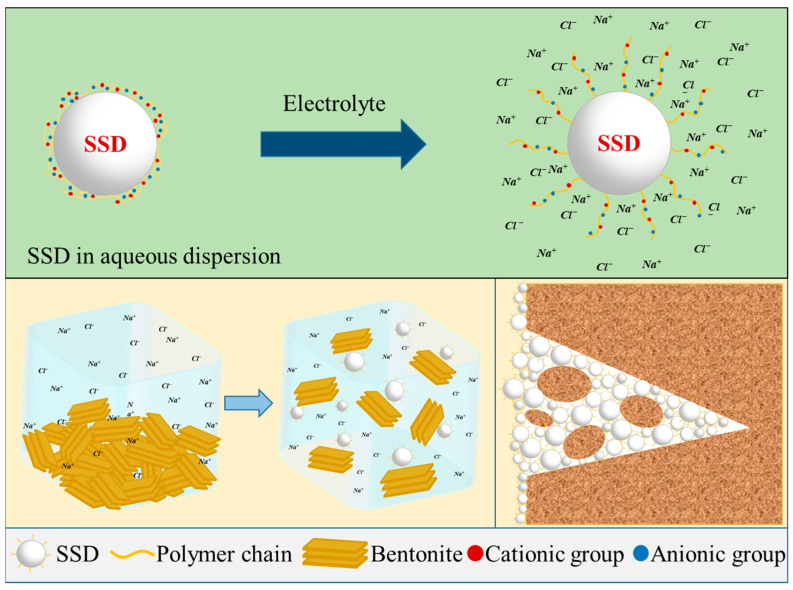
Mechanism of SSD in drilling fluid.

The mechanism of SSD in drilling fluids was revealed through a series of experiments, as shown in Figure 11. SSD is an amphoteric ion-modified styrene-based micro-sphere, in which the surface polymers are curled due to the polyelectrolyte effect. When SSD is in the aqueous dispersion system, it exhibits salt-responsive properties with the added electrolytes, such as NaCl, and the polymer chains on its surface gradually stretch, leading to an increase in the visible light transmittance of the aqueous dispersion of SSD. Therefore, when SSD is added to the drilling fluid, it can improve the dispersion of clay under salty conditions and improve the stability of the drilling fluid, which in turn reduces the fluid loss of the drilling fluid and enhances its rheological properties. After SSD enters the formation with the drilling fluid, it can also be the first to enter and plug the microporosity and microcracks under the effect of differential pressure, preventing the drilling fluid from intruding into the formation, and maintaining the stability of the wellbore and drilling safety.

## 4. Conclusions

(1) A new amphoteric ion-modified, styrene-based nano-microsphere, SSD, was developed with a particle size of 228 nm.

(2) SSD shows salt-responsive properties; its surface molecular chains gradually stretch and solution transparency increases with increasing salinity, thus providing a better ability to plug rock pores.

(3) Compared to general styrene-based microspheres modified with anions, SSD has an advantage in improveding the performance of drilling fluids at high-temperature and high-salinity conditions. It can better synergize with clay to improve the suspension and dispersion stability of the drilling fluid, improve the quality of the mud cake, and reduce fluid loss.

## Data Availability

Data available on request due to privacy.
